# Epidemic dispersion of HIV and HCV in a population of co-infected Romanian injecting drug users

**DOI:** 10.1371/journal.pone.0185866

**Published:** 2017-10-09

**Authors:** Simona Paraschiv, Leontina Banica, Ionelia Nicolae, Iulia Niculescu, Adrian Abagiu, Raluca Jipa, Andrea-Clemencia Pineda-Peña, Marta Pingarilho, Emil Neaga, Kristof Theys, Pieter Libin, Dan Otelea, Ana Abecasis

**Affiliations:** 1 Molecular Diagnostics Laboratory, National Institute for Infectious Diseases ‘Matei Bals’, Bucharest, Romania; 2 Clinical Department, National Institute for Infectious Diseases ‘Matei Bals’, Bucharest, Romania; 3 SMZ Süd—Kaiser-Franz-Josef-Spital, 4. Med. Abteilung, Vienna, Austria; 4 Global Health and Tropical Medicine, Instituto de Higiene e Medicina Tropical, Universidade Nova de Lisboa, Lisbon, Portugal; 5 Molecular Biology and Immunology Department, Fundación Instituto de Inmunología de Colombia (FIDIC) and Basic Sciences Department, Universidad del Rosario, Bogotá, Colombia; 6 KU Leuven—University of Leuven, Department of Microbiology and Immunology, Rega Institute for Medical Research, Clinical and Epidemiological Virology, Leuven, Belgium; 7 Artificial Intelligence lab, Department of computer science, Vrije Universiteit Brussel, Brussels, Belgium; Fudan University, CHINA

## Abstract

Co-infections with HIV and HCV are very frequent among people who inject drugs (PWID). However, very few studies comparatively reconstructed the transmission patterns of both viruses in the same population. We have recruited 117 co-infected PWID during a recent HIV outbreak in Romania. Phylogenetic analyses were performed on HIV and HCV sequences in order to characterize and compare transmission dynamics of the two viruses. Three large HIV clusters (2 subtype F1 and one CRF14_BG) and thirteen smaller HCV transmission networks (genotypes 1a, 1b, 3a, 4a and 4d) were identified. Eighty (65%) patients were both in HIV and HCV transmission chains and 70 of those shared the same HIV and HCV cluster with at least one other patient. Molecular clock analysis indicated that all identified HIV clusters originated around 2006, while the origin of the different HCV clusters ranged between 1980 (genotype 1b) and 2011 (genotypes 3a and 4d). HCV infection preceded HIV infection in 80.3% of cases. Coincidental transmission of HIV and HCV was estimated to be rather low (19.65%) and associated with an outbreak among PWID during detention in the same penitentiary. This study has reconstructed and compared the dispersion of these two viruses in a PWID population.

## Introduction

The use of contaminated needles is an effective way of spreading HIV; it accounts for 10% of infections worldwide and for more than 40% in some regions and countries (Eastern Europe, South America, and East and Southeast Asia) [1, 2]. Seroprevalence of HCV antibodies is very frequent among people who inject drugs (PWID) rising up to 90%; in the general HIV infected population it raises only to about 10% [3–5]. HIV-HCV co-infection affects the natural history of both diseases: increased mortality risk, increased AIDS and liver-related disease mortality, accelerated hepatic fibrosis that leads to cirrhosis and/or hepatocellular carcinoma [6].

Both viruses have a high level of genetic diversity within and between hosts and a high rate of evolution. For HIV-1, several groups, subtypes and circulating recombinant forms (CRFs) are described; for HCV seven different genotypes have been described and each genotype has distinct subtypes [7, 8]. The high evolutionary rate allows us to study the transmission history of both viruses by analysing their genomic sequences with phylogenetic methods.

In recent years, an important HIV outbreak was reported among PWID in Romania, with 30.6% of the total newly diagnosed HIV cases in the country in 2012 [9]. The most affected region was Bucharest and its suburbs where more than 90% of Romanian PWID are living [10]. The Romanian PWID infected with HIV are mainly young males, unemployed, often diagnosed in penitentiary, who inject both heroin and new psychoactive drugs („legal highs”), and are frequently co-infected with HCV. Two predominant HIV strains were identified in PWID: subtype F1 which had already been circulating in sexual and nosocomially infected patients and the newly endemic CRF14_BG, most probably originating in Spain or Portugal [11, 12].

Previous studies established the role of phylogenetic analysis in assessing and characterizing transmission networks within particular populations, but these were mainly performed in specific populations of HIV patients. However, few studies have been performed that reconstruct simultaneously the transmission history of HIV and HCV viruses in the same population of HIV-HCV co-infected patients [13, 14] and none analysed the overlap between HIV and HCV transmission networks. Our objective was to reconstruct the history and the transmission patterns of HIV and HCV, to assess the likelihood of simultaneous transmission of these two viruses in a population of co-infected PWID and to analyse the congruence between HIV and HCV transmission networks.

## Materials and methods

### Study population

We have included in this study a number of 117 injecting drug users who were diagnosed with HIV infection between 2011 and 2014 in the National Institute for Infectious Diseases ‘Matei Bals’, the reference center for HIV genotyping. All patients were HCV co-infected and naive to antiretroviral treatment. A number of 35 non-PWID, HCV mono-infected patients, were also included in the study.

Blood samples and clinical and epidemiological data were collected through a questionnaire survey. Laboratory testing (i.e. HIV viral load, CD4 count, HCV serology, HCV viral load) was performed with *in vitro* diagnostic (IVD) tests.

### Ethics statement

The study was conducted according to the Declaration of Helsinki and approved by the Ethical Committee of the National Institute for Infectious Diseases ‘Matei Bals’. All the subjects included in this study were adults who provided their written informed consent.

### HIV genotyping

Total viral RNA was extracted from 1 ml of plasma using the automated NucliSens EasyMAG nucleic acid extraction system (BioMerieux, Marcy-l'Étoile, France), according to the manufacturer's instructions and eluted in 25μl of water. Ten microliters of RNA were used for HIV genotyping. Reverse transcription and amplification of the HIV-1 *pol* gene (1301 nucleotides, HXB2: 2253–3554) were performed using the Viroseq HIV-1 Genotyping System (Celera Diagnostics, Alameda, CA) and sequenced with the ABI 3500 Genetic Analyzer (Applied Biosystems, Foster City, CA). The primary data were analysed using Sequencing Analysis Software Version 3.7 (Life Technologies) and the generated sequences were assembled with ViroSeq 2.8 HIV-1 Genotyping System Software (Celera Diagnostics, Alameda, CA). REGA HIV-1&2 automated subtyping tool version 3.0 was used to assign the HIV-1 subtype [15]. Unclassified samples were further analysed in order to identify possible recombination breakpoints using Simplot v3.5.1 software and the Los Alamos HIV-1 subtype reference dataset (sliding window: 400-nt, T:t ratio = 2.0, model of evolution: Kimura two-parameter, bootstrap: 1000 replicates). All sequences were screened for hypermutation using the Hypermut 2.0 algorithm [16].

### HCV genotyping

Following RNA extraction as reported above, the NS5b and NS3 regions were sequenced for HCV genotyping. Ten microliters of extracted viral RNA were used for reverse transcription with Transcriptor Reverse Transcriptase (Roche) and random primers (Roche). For amplifying the NS5b region (374 nucleotides, H77: 8256–8630), 1,1X Platinum PCR SuperMix (Life Technologies) and previously published primers were used [17]. Different sets of primers, specifically designed for genotype 1, 2, 3a, 3b and 4 and 1,1X Platinum PCR SuperMix (Life Technologies) were used to amplify the first part of the NS3 gene (710 nucleotides, H77:3326–4036) [18].

The NS5b and NS3 amplicons were bidirectionally sequenced using BigDye® Terminator system v 1.1 (Life Technologies) and ABI 3500 Genetic Analyzer (Applied Biosystems). Seqscape version 2.7 (Applied Biosystems) was used to assemble and generate consensus sequences. Genotyping was done using the publicly available algorithm Oxford HCV Automated Subtyping Tool (Version 2.0) [19].

### Phylogenetic, phylodynamic analysis and divergence times of transmission networks

Reference sequences were selected from public databases [20, 21] taking into account genotype / subtype, genomic region and geographic origin. To retrieve control sequences for transmission cluster reconstruction, the most similar HIV sequences were retrieved from our and Los Alamos database using BLAST. Duplicates and clones were discarded. Of 421 selected control sequences, those representative to geographical and time dispersion of the patients sample, were subsequently used for each analysis. Controls for all the HIV-1 subtypes and CRFs identified among Romanian PWID were selected as follows: CRF14_BG (Spain, Portugal, Romania), subtype F1 (Angola, Romania), subtype B (European countries), CRF35_AD (Afghanistan, Iran). CRF14BG epidemics from Spain-Portugal [11] and Greece [12] were previously linked to the Romanian HIV epidemic among PWID. Subtype F1 strains from Romanian PWID were previously shown [11] to originate from strains already circulating in Romania and formerly described as related with strains from Angola [22, 23].

A similar procedure was used for the HCV dataset, where a number of 304 control sequences were used. Multiple alignments were generated using Muscle [24].When available, concatenate alignments of NS5b and NS3 sequences were used in the Bayesian analyses.

Phylogenetic analyses were performed using both maximum-likelihood (ML) and Bayesian approaches, after selecting the best-fit nucleotide substitution model using JModelTest (GTR+4Γ+I for HIV-1 and TN93 for HCV). Datasets for each HIV-1 subtype and HCV genotype identified among Romanian PWID were constructed and analyzed separately. ML trees were inferred using FastTree software [25] and Bayesian phylogenetic analyses were performed with BEAST version 1.8.1 [26] using a Bayesian Skyline coalescent tree prior [27] and the uncorrelated lognormal relaxed clock model [28]. Two Markov Chain Monte Carlo (MCMC) runs were computed separately for 10^7^ generations with a burn-in of 10%. The output of the MCMC analysis was tested for convergence by means of effective sampling size (ESS>100) using the program Tracer v1.6 [29]. The maximum clade credibility (MCC) tree was summarized with TreeAnnotator program [30].

Transmission clusters were identified as clusters in the ML phylogenetic tree with SH-like support higher or equal to 0.9 and were further confirmed with Bayesian phylogenetic analyses if supported by a Bayesian posterior probability value of 1.0 [31, 32]. The temporal signal of both datasets was evaluated with TempEst [33]. Data were statistically evaluated and plotted using GraphPad Prism v6.0. The Mann-Whitney test was performed to identify differences between groups and the Chi-square or Fisher exact test were used to evaluate the distribution in particular subgroups; p-values <0.05 were considered significant.

## Results

### Characteristics of the population

The population of 117 co-infected PWID included in this study were diagnosed with HIV between 2011 and 2014. The diagnosis of HCV and HIV was simultaneous for 71 patients (60.7%), while for the remaining 46 individuals HCV diagnosis was established earlier than the HIV diagnosis. Most of the PWID (64.1%, 75) were asymptomatic at the HIV diagnosis, while 35.9% were diagnosed in more advanced HIV infection stages, classified as stage B (27%, 32) and C (9%, 10) according to CDC and WHO Staging Systems [34]. The 35 control HCV mono-infected patients were diagnosed during the same period of time, also in the National Institute for Infectious Diseases ‘Matei Bals’.

### Disease severity differs when comparing patients infected with HIV-1 subtype F1 and with CRF14_BG-like strains

**Fig 1:**
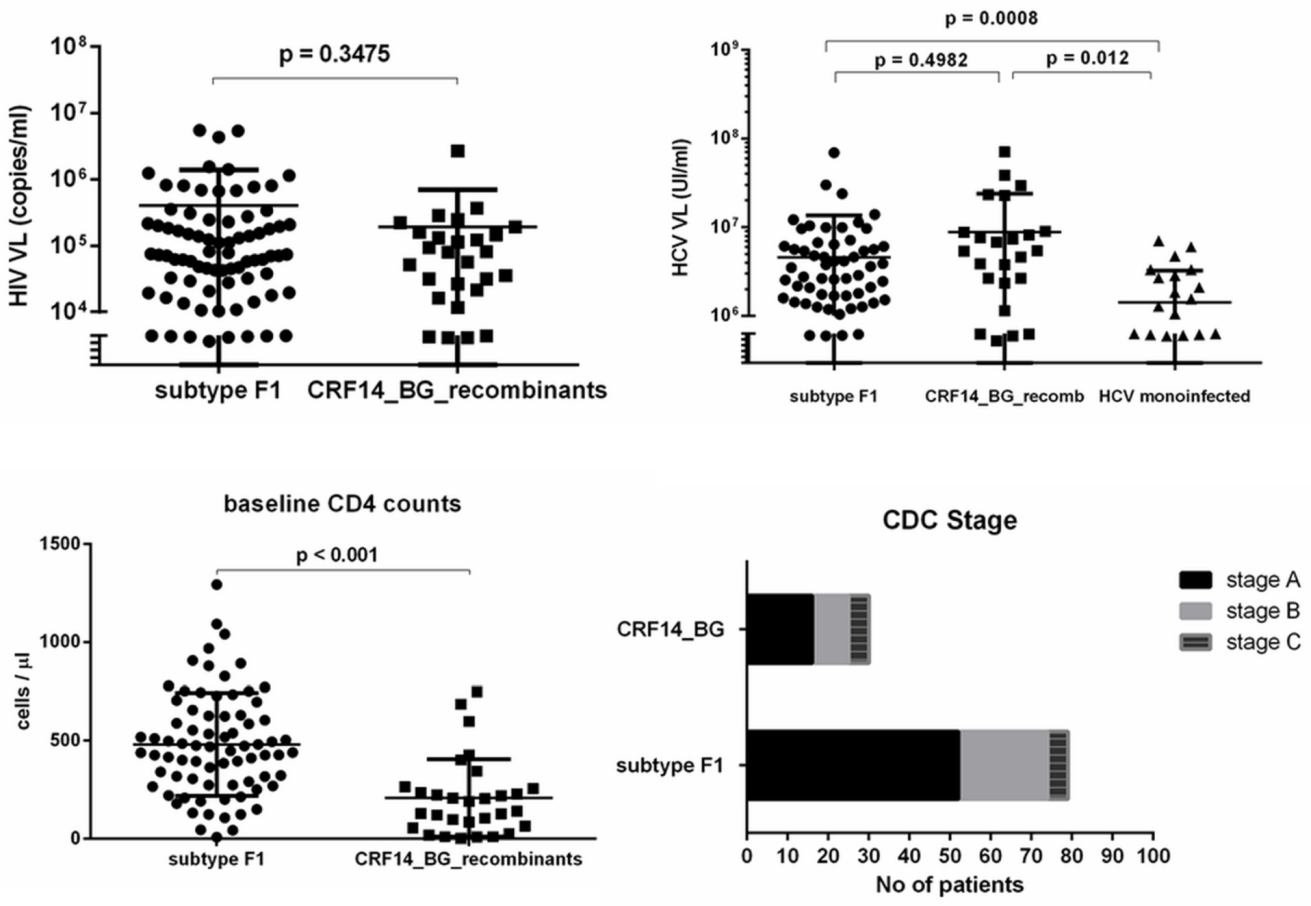
Clinical and laboratory characteristics of studied patients. A. Differences in HIV-VL among PWID, the two groups infected with F1 subtype and recombinant forms (CRF14_BG, URFs of CRF14_BG and F1) B. Differences in HCV-VL in mono-infected and HIV co-infected patients (PWID) C. CD4 count distribution in PWID at baseline D. Clinical HIV stages at diagnosis in PWID.

HIV-1 subtype analysis showed that 67.5% (n = 79) of the patients were infected with subtype F1 viruses; recombinant forms present in a significant proportion (26%; n = 30) were CRF14_BG (n = 19, 16.24%) and unique recombinant forms (URF) of CRF_14BG and F1 (n = 11, 9.4%). The PWID infected with these recombinant forms had significantly lower CD4 counts at baseline than patients infected with subtype F1 strains. It is true, though, that an estimation of the time to the infecting event was not possible in either case. No significant differences between these two groups were observed when HIV viral load (VL) or CDC stages were analysed (Fig 1). However, a higher percentage of patients infected with CRF14_BG were diagnosed at stages B and C than the group infected with F1 (47% vs. 34%). Few patients (n = 8, 6.8%) were infected with other types of HIV: subtype B, CRF35_AD and URFs between subtype A1 or B and F1 (Table 1).

**Table 1:** Distribution of HIV subtypes and HCV genotypes in Romania co-infected PWID. Chi-square analysis was performed on 2x3 contingency table with pure HIV subtype /recombinant forms and HCV genotype 1a/1b/other and showed no significant difference in HIV subtype and HCV genotype distribution (p = 0.784).

HIV subtypes	HCV genotypes					Total
	1a	1b	3a	4a	4d	
Subtype F1	30	29	9	6	5	**79**
Subtype B	3	1	0	0	0	**4**
CRF14_BG	7	8	2	0	2	**19**
CRF35_AD	1	0	0	0	0	**1**
Recombinants CRF14_BG and F1	5	2	2	1	1	**11**
Other recombinants	2	0	1	0	0	**3**
**Total**	**48**	**40**	**14**	**7**	**8**	**117**

### HCV viral loads are higher in patients with HIV co-infection

All 117 patients were tested to assess the HCV genotype by analysing NS5b and/or NS3 regions of HCV. Both NS5b and NS3 sequences were available for 110 patients, while for the remaining patients only the NS5b region was available. The genotype assessment indicated that the majority of PWID were infected with genotype 1a (n = 47, 40%) and 1b (n = 40, 34%), but genotypes 3a and 4 (a, d) were also identified (Table 1). HCV subtype assignment was concordant in the two genomic regions in the majority of cases (n = 108, 98.2%), with only two exceptions of genotype 1 sequences (2749bh2013 and 2727bh2013) assigned as 1a in NS3 and 1b in NS5b. Unlike HIV-HCV co-infected PWID, the individuals in the HCV mono-infected group were exclusively infected with genotype 1b strains. Significant differences (Fig 1) in HCV VL were observed when comparing mono-infected with co-infected patients, with higher viral loads in the group of co-infected.

### All identified HIV clusters are large and originated around 10 years ago

**Fig 2:**
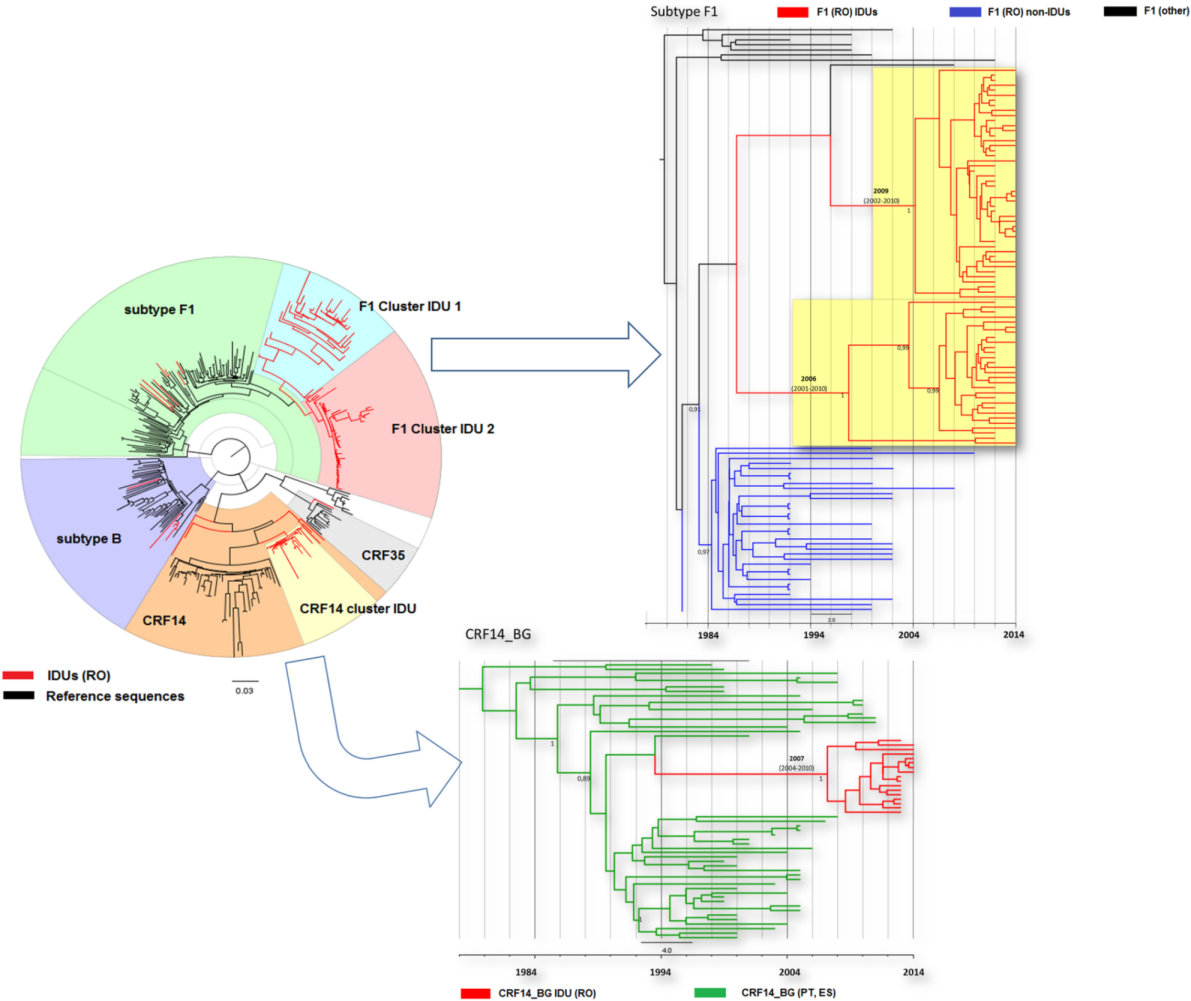
Phylogenetic analysis of HIV-1 Romanian PWID sequences. Circular tree: Maximum likelihood tree generated as described under Methods section. The sequences corresponding to PWID are colored in red and in black the control sequences. Rectangular trees: The transmission networks identified in PWID were further confirmed with Bayesian phylogenetic analyses. Molecular clock analyses were performed separately for F1 subtype and CRF14_BG sequences. In F1 subtype tree, in red are marked the PWID sequences, in blue are represented sequences of sexually infected patients from Romania and in black are marked F1 sequences from Angola. In CRF14_BG tree, the PWID sequences in red are marked and in green the sequences from Spain and Portugal (PWID). Different clusters were highlighted on the trees and tMRCA was specifically marked for each cluster. The posterior probability support for each transmission cluster is represented at the internal nodes. The scale is in years.

Phylogenetic analysis of Romanian PWID using control sequences from Romania and other parts of the world showed that 90 HIV sequences (76.9%) were part of well-supported phylogenetic clusters, suggesting high levels of transmission networking. Three major HIV local transmission networks were identified by phylogenetic analysis, two within subtype F1 and one with CRF14_BG (Fig 2). One of the subtype F1 clusters included 29 PWID patients, 24 men and 5 women; 17 men (58%) were diagnosed in the same penitentiary. The other subtype F1 cluster identified in PWID is larger and consists of 44 sequences corresponding to 34 men and 10 women living in the same geographical area. The lineages of both F1 clusters seem to originate from strains previously identified in Romanian people living with HIV (PLWH). The transmission network identified in PWID infected with CRF14_BG involved 17 subjects, diagnosed with low CD4 counts (mean 138 cells/mm). The CD4 counts were significantly lower than in patients from the two F1 transmission networks (mean CD4 count of cluster 1–430 cells/mm, cluster 2–510 cells/mm, p<0.001).

Phylodynamic analysis for all three HIV transmission networks among PWID indicated similar starting time points, dating back to around 10 years ago (2006–2009, widest %95 HPD: 2001–2010). The results are in concordance with the epidemiological and seroconversion data available from these patients.

### HCV transmission clusters are smaller and their origins are more dispersed in time

**Fig 3:**
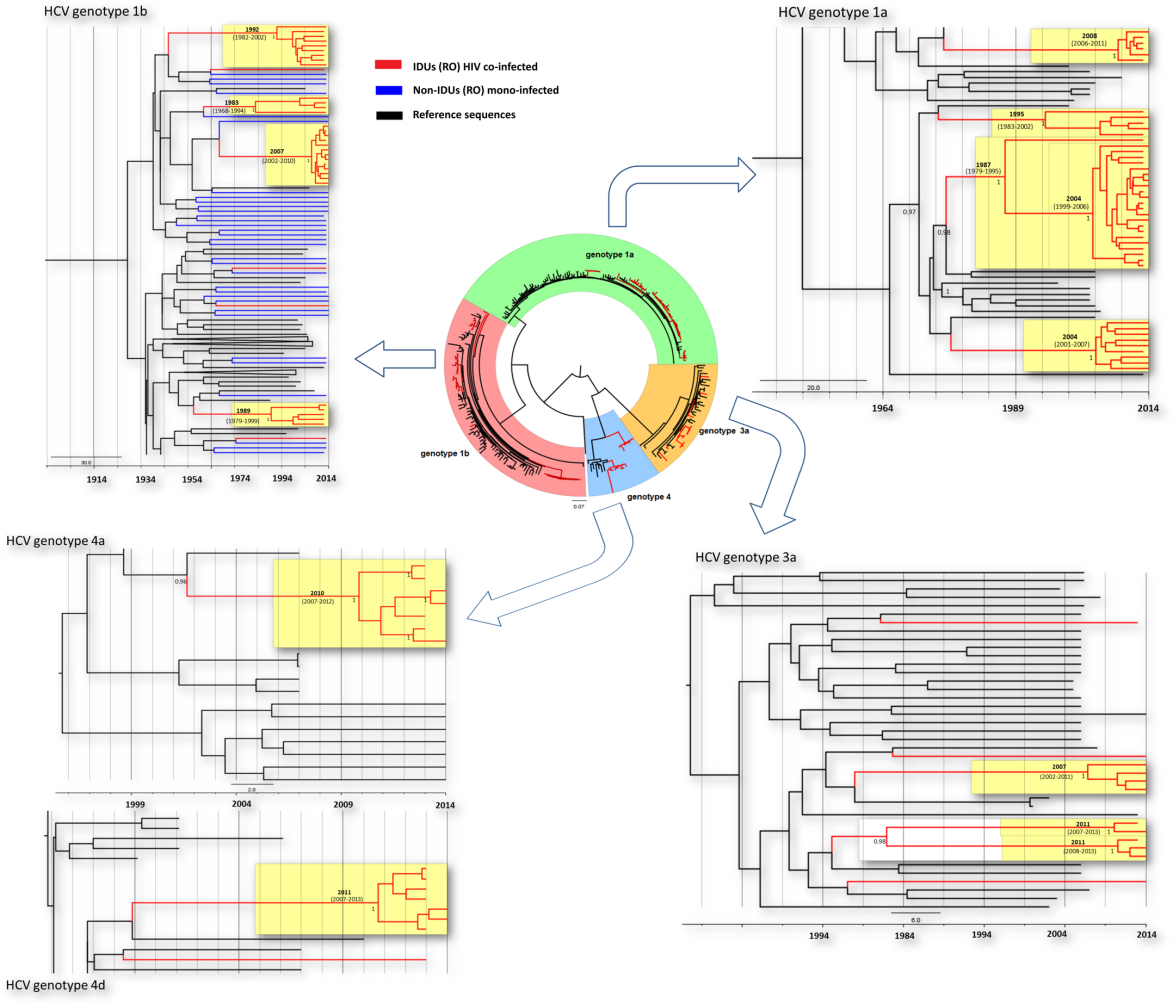
Phylogenetic analysis of HCV sequences in Romanian HIV co-infected PWID. Circular tree: Maximum likelihood tree generated as described under Methods section. The sequences corresponding to PWID are colored in red and in black the control sequences. Rectangular trees: The transmission networks identified in PWID were further confirmed with Bayesian phylogenetic analyses. Molecular clock analyses were performed separately for HCV genotypes 1a, 1b, 3a, 4a and 4d. Different clusters were specifically indicated on the trees. The posterior probability support and tMRCA for each transmission cluster are represented at the internal nodes. The scale is in years.

Phylogenetic reconstructions uncovered several HCV transmission clusters presented in Fig 3. As opposed to HIV where the virus has spread in only three large clusters, HCV dispersion occurred through multiple transmission networks. In total, 97 HCV sequences (82.9%) clustered in 13 transmission clusters. In comparison, within the HCV mono-infected control group only 8 out of 33 (24.2%) HCV sequences were found to form phylogenetic clusters (χ^2^ = 42.18, p<0.001).

Four well supported clusters of different sizes were observed in both genotypes 1a and 1b; the largest consisted of 23 sequences (genotype 1a) and 13 sequences (genotype 1b) (Table 2). Three small genotype 3a clusters were observed, while genotype 4a and 4d sequences identified among PWID formed each one single well supported cluster (Fig 3).

**Table 2:** HCV and HIV transmission networks identified within Romanian PWID.

HCV genotype	Cluster	Number of PWID sequences within the cluster	(%)	Sampling period	tMRCA (95% HPD)
**Genotype 1a (N = 48)**	a	6	12.5	2013–2014	**2008** (2006–2011)
	b	5	10.42	2013–2014	**1995** (1983–2002)
** **	c	23	47.92	2013–2014	**1987**(1979–1995)
** **	d	9	18.75	2013–2014	**2004** (2001–2007)
	**Total**	**43**	**89.58**		
**Genotype 1b (N = 40)**	e	9	22.50	2013–2014	**1992** (1982–2002)
** **	f	4	10.00	2013–2014	**1983** (1968–1994)
	g	13	32.5	2013–2014	**2007** (2002–2010)
** **	h	5	12.50	2013–2014	**1989** (1979–1999)
	**Total**	**31**	**77.5**		
**Genotype 3a (N = 14)**	i	4	28.57	2013–2014	**2007** (2002–2011)
** **	j	2	14.29	2013–2014	**2011** (2007–2013)
	k	3	21.43	2013–2014	**2011** (2008–2013)
	**Total**	**9**	**64.28**		
**Genotype 4a (N = 7)**	l	7	100	2013–2014	**2010** (2007–2012)
**Genotype 4d (N = 8)**	m	7	87.5	2013–2014	**2011** (2007–2013)
**HIV subtype**	** **	** **	** **	** **	** **
**Subtype F1 (N = 79)**	1	29	36.71	2013–2014	**2006** (2001–2010)
** **	2	44	55.70	2013–2014	**2009** (2002–2010)
	**Total**	**73**	**92.40**		
**CRF14_BG and recombinants (N = 30)**	3	17	56.67	2013–2014	**2007** (2004–2010)

**Fig 4:**
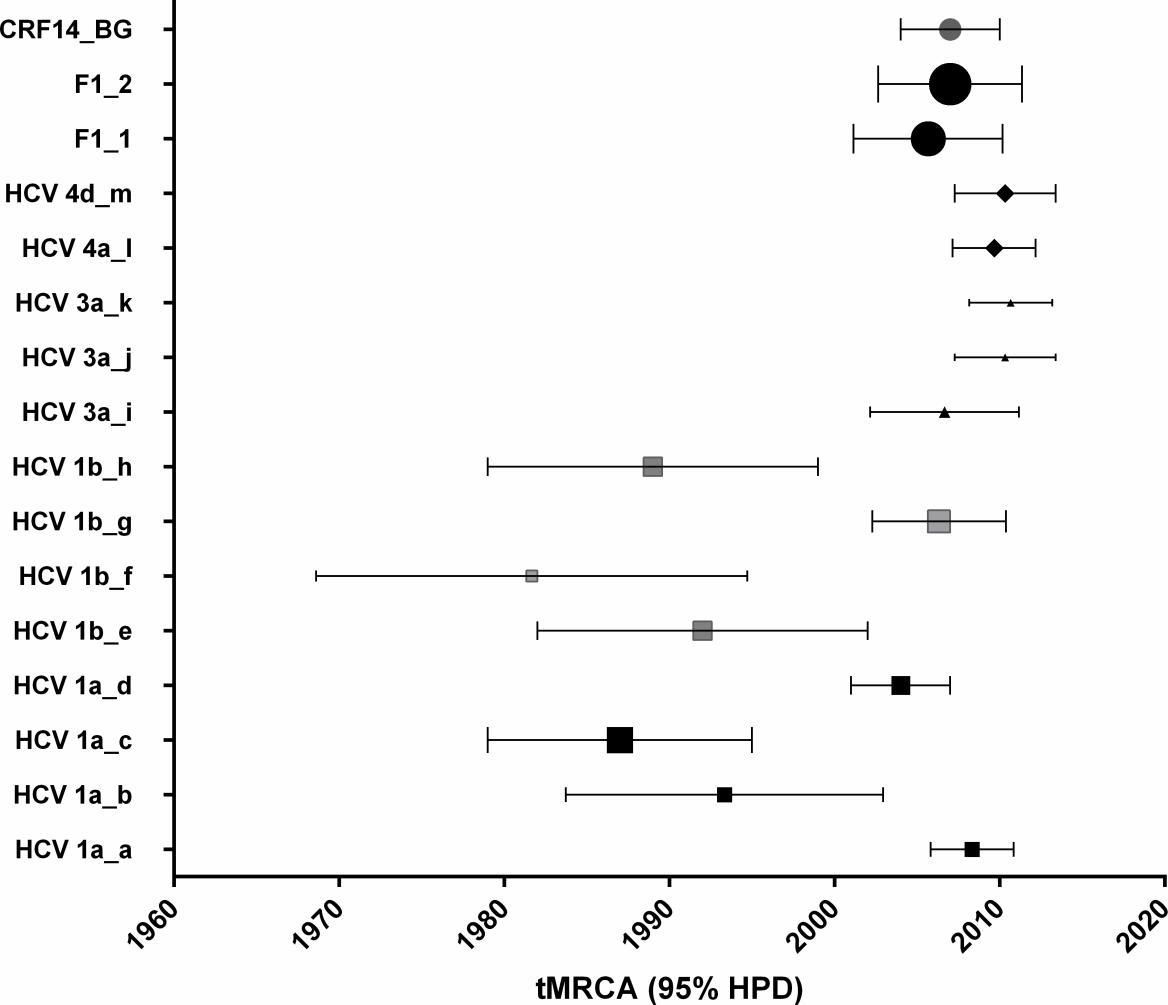
The most recent common ancestor (tMRCA) of HIV and HCV transmission clusters in PWID. The mean tMRCA and the high posterior density interval (95%HPD) for each identified transmission cluster are plotted.

Molecular clock analysis of HCV transmission clusters revealed important differences in origin and starting time points. The differences in divergence time were observed within as well as among genotypes. Based on relaxed clock and constant size population model implemented in a Bayesian MCMC framework, the oldest PWID network belongs to genotype 1b and originated around 1983 (95%HPD: 1968–1994), followed by the larger HCV genotype 1a cluster, dated back to 1987 (Figs 3 and 4). However, the time to the most recent common ancestor (tMRCA) estimated for a sub-group of this 1a cluster consisting in 22 out of 23 sequences was more recent (2004, 95%HPD 1999–2006).

Genotype 1b was found to be the first introduced in the studied population: the oldest cluster was estimated to be 30 years old in PWID and more than 50 years in non-PWID. Genotypes 3 and 4 are the most recently introduced in this risk group population, dating back 2–3 years before sampling. The divergence times for all important PWID networks, HIV and HCV, are presented in Table 2 and graphically illustrated in Fig 4.

### Coincidental transmission of HIV and HCV is uncommon and occurred in 19.65% of the patients

**Fig 5:**
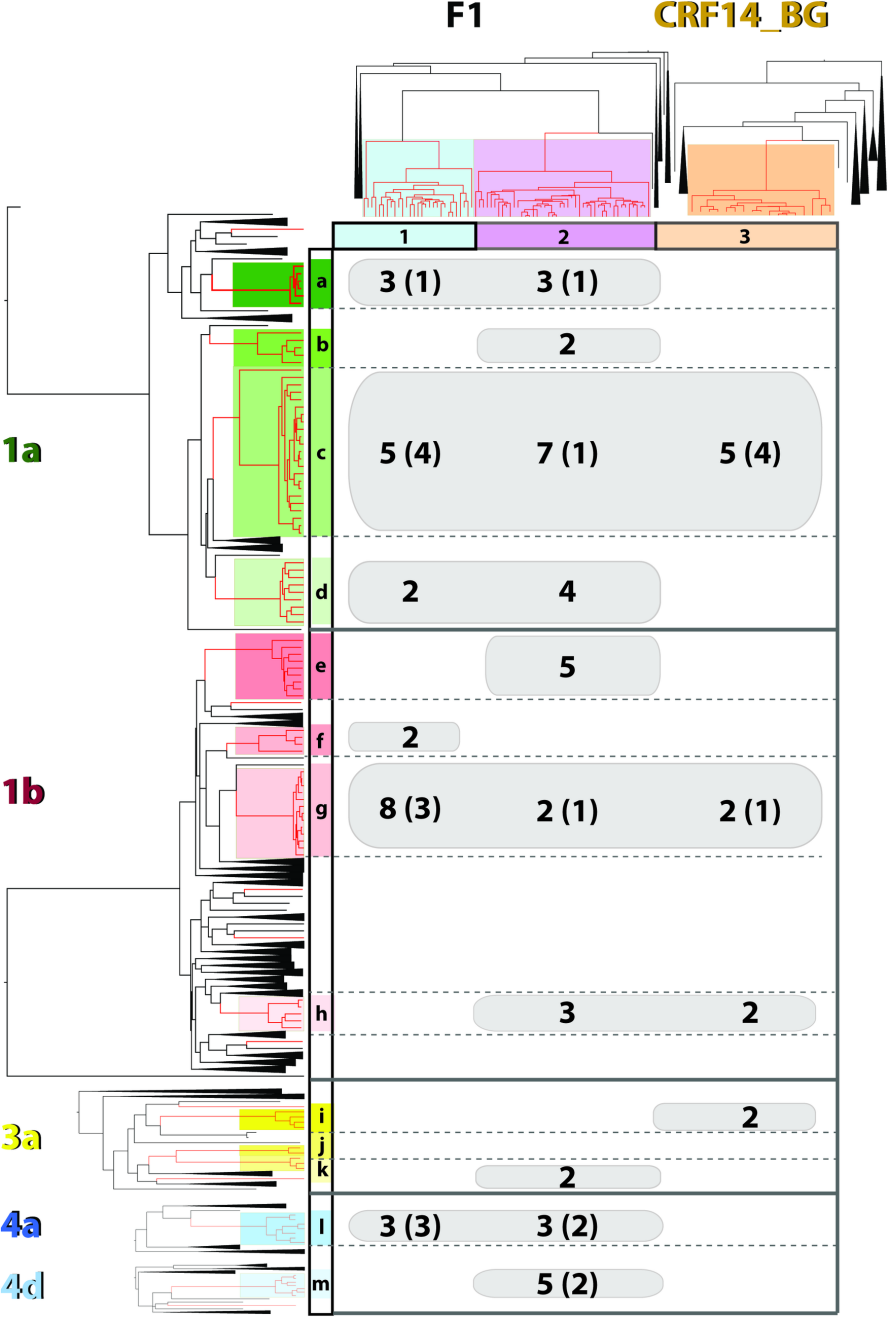
Transmission dynamics of HIV and HCV infections. The comparative analysis of the clustering patterns in HIV-HCV co-infected patients is presented. The number of all sequences that were part of both HIV and HCV clusters are shown, in brackets being the simultaneous transmission cases; sequences with a difference of mean divergence times between HCV and HIV (ΔtMRCA) <1.

We have comparatively analysed the patterns of patients distribution in HIV and HCV transmission clusters (Fig 5). Eighty patients were present both in HCV and in HIV transmission clusters (68.4% of all co-infected patients; 82.5% and 88.9% of all HCV and HIV patients present in clusters, respectively). Furthermore, 70 patients (59.8%) were present in the same HIV and HCV cluster with at least one other patient, indicating transmission of HIV and HCV through (at least partly) overlapping transmission chains.

Phylogenetic analysis and, in particular, measuring the difference in divergence times between viral lineages within a co-infected individual has been proven useful in assessing the frequency of viral coincidental transmission in PWID in the absence of seroconversion data [35]. We have used the calculated difference of mean divergence times (ΔtMRCA) between HIV and HCV lineages within a patient to estimate the time period lapsed between the acquisition of the two viruses. Low ΔtMRCA indicates a higher likelihood that the two viruses might have been co-transmitted to a susceptible individual, while higher values of ΔtMRCA suggest that the viruses were transmitted at different and distinct time points.

For the majority of the patients this analysis suggested that HCV was introduced first, followed by HIV. However, for 5 patients, HIV was introduced first; all of them were infected with subtype F1 and genotype 3a or 4 HCV strains. Twenty three patients had ΔtMRCA between HIV and HCV lineages of less than one year, suggesting potential co-transmission of HIV and HCV. These 23 patients (19.65%) were present in the same HIV and HCV cluster with at least one other patient, suggesting a potential simultaneous transmission (co-transmission) of both infections. We also found that such transmission events are largely associated with particular HCV transmission clusters, specifically clusters c and l (Fig 5). Going back to the records of these patients, we found that twenty-two of those 23 patients were in detention at the moment of study enrolment. This population has a statistically significant higher probability of coincidental transmission (p<0.0001) when compared to the rest of the patients. Five of these patients were infected with CRF14_BG and the rest with subtype F1; the infective HCV genotypes were 1 or 4 (Fig 5).

## Discussion

The PWID represent no more than an estimated 0.2–0.5% of the world’s population, but they account for approximately 5–10% of all people living with HIV and 6.8% of the persons infected with HCV [36, 37]. Since 2008 injected amphetamine type stimulants („legal highs”) became available on the market in Romania and were low-priced. As a result, the number of PWID increased dramatically in recent years so that Romania reported an HIV outbreak among PWID that started in 2011; more than 90% of PWID being concentrated in Bucharest and its surroundings [10, 38]. Numerous people who were injecting heroin have switched to the new class of drugs due to the lower price and because they were legal at that time. An important behavioral characteristic is that the latter require up to 10 injections every day. This fact, which was coincident with an interruption in needle exchange programs, greatly impacted the needle-sharing behavior in the country [39]. Preliminary analyses indicated the circulation of CRF14_BG strains in PWID, HIV strains associated with lower CD4 counts and X4 tropism at baseline, as compared with the subtype F1 infected PWID [11, 40].

In this study we have identified, characterized and compared the viral transmission networks of HIV and HCV in a population of HIV-HCV co-infected PWID–using a group of HCV mono-infected patients as controls—and we evaluated the time of introduction of these viruses in this population and explored the probability of co-transmission of the two infections.

The clinical and epidemiological impact of HIV-HCV co-infections in PWID has been described by several studies [6, 41], but only one analysed in parallel the evolutionary history of these two viruses within this particular risk population [35]. Viral genetic information expressed as nucleotide sequences and analysed with phylogenetic and population genetics has been shown to be very useful in identifying and characterizing transmission clusters of rapidly evolving viruses such as HIV and HCV [42, 43].

Phylogenetic analysis performed in this study showed high levels of transmission networking among Romanian PWIDs: three HIV clusters and thirteen HCV clusters have been identified. The majority of HIV strains fell in two F1 clusters that were nested within Romanian reference strains. This suggests that F1 strains circulating for a long time among Romanian patients, infected sexually and nosocomially were the origin of the subtype F1 outbreaks in PWID and that these strains were restricted to Romania. The CRF14_BG strains circulating in PWID are related with those described earlier in Spain and Portugal in the same risk group population [11]. However, due to the limited number of CRF14_BG sequences available in public databases, direct filiation between the two epidemiologic events cannot be ascertained. Previous results indicated that the CRF14_BG sequences from Romanian PWID were closely related with the Greek PWID sequences and formed a single monophyletic cluster. Molecular clock analysis revealed that CRF14_BG strains circulating in PWID from Athens originated from one or several strains circulating in Bucharest [12]. Our phylodynamic analyses indicated that all HIV transmission networks originated at similar time points, dating back 7 to 10 years ago, coincident with the availability of these new injectable amphetamine type stimulants on the market. The patients infected with CRF14_BG had significantly lower CD4 counts at baseline than those infected with F1 subtype strains despite the fact that phylogenetic and seroconversion data suggest their being infected at similar time points. Although we have included in our analysis all the PWID presented in our clinical settings during this period of time, the number of patients infected with CRF14_BG recombinants was smaller than those infected with subtype F1 and this might have impact on the statistical analysis. However, these data are in agreement with previous studies showing an association between CRF14_BG infection, CD4 T cells decline and disease severity, characterized by CXCR4 usage [11, 40, 44].

On the other hand, regardless of the HIV infecting strain, HCV viral loads were significantly higher in co-infected PWID than in HCV mono-infected patients. This difference does not seem to be related to the HCV genotype. Moreover, when comparing HCV mono-infected patients with co-infected PWID carrying HCV genotype 1b, the difference in viral load continued to be statistically significant (S1 Fig). Several studies have shown an accelerated disease progression in HIV-HCV co-infected patients, HIV infection increasing the HCV replication, hepatic inflammation and impairing HCV-specific immune response [6]. Since CRF14_BG induced a massive depletion of CD4 T cells it was expected to be associated with impaired HCV immune control and thus higher replication and hepatic fibrosis. However, no significant difference in HCV VL was observed between CRF14_BG and subtype F1 infected patients or between HCV VL and CD4 count. Possibly, longer infection periods could be needed to impair the established HCV-specific immune response, since in most of the cases, HCV infection preceded HIV infection.

Previous studies have shown the genotype 1b to be mainly acquired through blood transfusion, while genotypes 1a and 3a are associated with intravenous drug use [45]. In other European countries, mostly HCV genotype 4 has been identified in PWID: subtype 4a in Greece, 4d in Italy and The Netherlands and 4c and 4d in Spain were linked to drug usage [46–49]. Our results indicate the circulation of multiple HCV genotypes in co-infected Romanian PWID: 1a, 1b, 3a, 4a and 4d. However, only HCV genotype 1b was found in the HCV mono-infected group, indicating a higher complexity of the HCV epidemic among co-infected PWID.

According to phylodynamic analyses, HCV genotype 1b was the first to be introduced in Romania, followed by genotype 1a. The oldest 1b cluster was estimated to be 30 years old in PWID and more than 50 years in non-PWID. Previous studies indicated that the circulation of this genotype in Romania was almost exclusive for a long period of time [50, 51]. Genotypes 3 and 4 are much more recent and linked to intravenous drug usage; with an estimated origin around 10 years ago, they might also be linked to the availability of injected amphetamine type stimulants [52].

The differences in transmission clustering between HCV and HIV might be explained in part by their estimated acquisition time. Previous observations based on classical epidemiological data indicated that HCV was introduced in PWID much earlier, being associated with heroin use while HIV infection is linked with new psychoactive drugs [11]. Phylodynamic analysis presented in this study sustains this observation, showing that HIV strains were introduced in PWID in recent years whereas HCV genotypes 1a and 1b had been circulating several decades before. However, HCV transmission networks of genotypes 3 and 4 apparently started later, as they were dated as very recent (2010–2011), thus indicating a potential linkage of these genotypes with new psychoactive drugs.

When comparing the transmission chains of HIV and HCV, we evaluated both the congruence between HIV and HCV transmission chains, as well as the likelihood of simultaneous transmission of both infections. We found that most patients (68.4%) were present both in HIV and HCV transmission clusters and that for the majority of the patients’ transmission of HIV and HCV occurred through (at least partly) overlapping transmission chains (Fig 5). We used the method reported by Ng TK and collaborators [35] to evaluate the frequency of viral coincidental transmission. Applying this method to Romanian co-infected PWID, we found that, for most of the patients, and in accordance with epidemiological data, HCV infection was acquired before HIV.

Nevertheless, we identified 23 cases of possible co-transmission (19.65%), corresponding to patients with ΔtMRCA<1 and clustering in partly overlapping transmission chains. Interestingly, these transmission events were associated with particular clusters of genotype 1a and 4a. Further investigation lead us to the finding that 22 of these 23 patients were in detention in the same penitentiary. While largely overlapping transmission chains would be expected, given the fact that this population forcely shares the same contact network while in detention, a higher likelihood of simultaneous transmission of the infections is not necessarily expected in this context. Therefore, our findings call for further investigation about what type of behavioral patterns explain the high rate of co-transmission of HIV and HCV in this penitentiary when compared to the rest of the PWID population. One potential bias in our analysis could be the subevaluation of possible super-infections and co-transmissions: phylogenetic analysis based on population sequencing considers only the predominant viral infective strain. Coincidental transmissions could be underestimated in our analysis if the transmitted strains did not become visible within the viral population.

The HIV epidemic among Romanian PWID continues to represent a major problem and HCV co-infection contributes to the worse prognostic within this specific risk population. This study clarifies and compares the patterns of spread and transmission of these two viruses in Romanian PWID, suggesting that a higher rate of transmission of HIV rather than HCV is associated with behavioral changes mainly following the availability of new types of injected drugs (‘legal highs’). While overlapping of HIV and HCV transmission chains is common, the estimated transmission of both viruses within a short time span is less common, with a rate of only 19.65% in this population, and largely associated with drug injection within the same penitentiary. Our results illustrate the need for better prevention policies in the PWID population, in particular and more urgently in the prison population, where an outbreak of simultaneous HIV and HCV transmissions seems to be occurring.

One limitation of the study is that the inmate population is overrepresented in the PWID so that extrapolation to the general population should be cautious. It should be though noted that, within this rather small group, no significant clinical or epidemiologic differences were noted according to the inprison vs. outprison status.

### Accession numbers

86 HIV nucleotide sequences reported in this paper were newly deposited in GenBank and have the following accession numbers: KX158994—KX159079. The remaining 31 HIV sequences were previously published in Genbank [11] and have the following accession numbers: KJ194665, KJ194822, KJ194819, KJ194811, KJ194662, KJ194666, KJ194821, KJ194824, KJ194733, KJ194816, KJ194818, KJ194826, KJ194668, KJ194817, KJ194823, KJ194669, KJ194808, KJ194664, KJ194663, KJ194810, KJ194820, KJ194813, KJ194809, KJ194828, KJ194807, KJ194806, KJ194827, KJ194815, KJ194825, KJ194805, KJ194672.

HCV nucleotide sequences used in this analysis were recently deposited in Genbank. HCV-NS5b sequences were deposited with the following accession numbers: KX158877—KX158993. Accession numbers for HCV–NS3 nucleotide sequences corresponding to PWID are: KX159080—KX159189 and for the monoinfected patients (control group) are: KX159190—KX159224.

## Supporting information

S1 Fig HCV viral load (VL) comparison between HCV monoinfected and co-infected patients with genotype 1b. Statistical analysis was performed with Mann-Whitney test, p value < 0.05 was considered significant.
(TIF)
